# Analysis of IVF live birth outcomes with and without preimplantation genetic testing for aneuploidy (PGT-A): UK Human Fertilisation and Embryology Authority data collection 2016–2018

**DOI:** 10.1007/s10815-021-02349-0

**Published:** 2021-11-12

**Authors:** Kathryn D. Sanders, Giuseppe Silvestri, Tony Gordon, Darren K. Griffin

**Affiliations:** 1 School of Biosciences, University of Kent, Canterbury, CT2 7NJ UK; 2 CooperGenomics, London, UK

**Keywords:** Preimplantation genetic testing aneuploidy, Retrospective study

## Abstract

**Purpose:**

To examine the live birth and other outcomes reported with and without preimplantation genetic testing for aneuploidy (PGT-A) in the United Kingdom (UK) Human Embryology and Fertilization Authority (HFEA) data collection.

**Methods:**

A retrospective cohort analysis was conducted following freedom of information (FoI) requests to the HFEA for the PGT-A and non-PGT-A cycle outcomes for 2016–2018. Statistical analysis of differences between PGT-A and non-PGT-A cycles was performed. Other than grouping by maternal age, no further confounders were controlled for; fresh and frozen transfers were included.

**Results:**

Outcomes collected between 2016 and 2018 included total number of cycles, cycles with no embryo transfer, total number of embryos transferred, live birth rate (LBR) per embryo transferred and live birth rate per treatment cycle. Data was available for 2464 PGT-A out of a total 190,010 cycles. LBR per embryo transferred and LBR per treatment cycle (including cycles with no transfer) were significantly higher for all PGT-A vs non-PGT-A age groups (including under 35), with nearly all single embryo transfers (SET) after PGT-A (significantly more in non-PGT-A) and a reduced number of transfers per live birth particularly for cycles with maternal age over 40 years.

**Conclusion:**

The retrospective study provides strong evidence for the benefits of PGT-A in terms of live births per embryo transferred and per cycle started but is limited in terms of matching PGT-A and non-PGT-A cohorts (e.g. in future studies, other confounders could be controlled for). This data challenges the HFEA “red traffic light” guidance that states there is “no evidence that PGT-A is effective or safe” and hence suggests the statement be revisited in the light of this and other new data.

**Supplementary Information:**

The online version contains supplementary material available at 10.1007/s10815-021-02349-0.

## Introduction

Preimplantation genetic testing for aneuploidy (PGT-A) has utilised various genetic testing methodologies since 1993. The scientific rationale behind PGT-A is clear, to test for euploid embryos and preferentially transfer these euploids in order to improve in vitro fertilisation (IVF) outcomes. Its use however still remains controversial, particularly as to whether PGT-A improves IVF live birth rates [1, 2]. A number of PGT-A double-blinded, single-centre and multiple-centre randomised control trials (RCTs) have been performed, and subsequent meta-analyses have produced variable and contradictory reported benefits of PGT-A [3–5]. This is even after PGT-A technology used in RCTs had moved on to be able to measure aneuploidy in all 24 chromosomes [6–9].

In addition to RCTs, other authors have reported on PGT-A live birth outcomes from national IVF data collections. In 2015, Chang et al. published an analysis of 2011–2012 PGT outcomes from the US Society for Assisted Reproductive Technology (SART) Surveillance Data, comparing 5471 PGT-A cycles to 97,069 non-PGT-A IVF cycles, reporting increased odds of live-birth delivery (aOR 1.43; 95% CI, 1.26–1.62) per transfer for PGT-A patients of maternal age over 37 years (aOR 1.43), 35–37 years (aOR 1.13), but not for maternal age < 35 years (aOR 0.82) [10]. In 2020, Theobald et al. also reported on US SART and UK HFEA (Human Fertilisation and Embryology Authority) PGT-A data collections for the years 2014–2016 [11]. Although it should be noted that for this publication the authors were not able to differentiate between PGT-A, PGT for monogenic disorders (PGT-M) and PGT for structural rearrangement (PGT-SR) for the US SART data. This paper’s aim was to look at the incidence of PGT-A utilisation in IVF in these two countries, showing < 2% of IVF cycles were PGT-A in the UK and 13–27% were PGT-A in the USA. They reported that, in the UK, PGT-A cycles were roughly an equal mix of fresh and frozen embryo cycles, although they did not compare outcomes of PGT-A vs non-PGT-A cycles [11]. Theobald et al. also discussed many of the limitations of the HFEA PGT reporting system at the time of data collection. A number of UK single ART (assisted reproductive technology) centres have reported retrospective analysis of their PGT-A vs non-PGT-A outcomes [12, 13]. These centres have reported the individual centre outcomes although to date, no national study of PGT-A outcomes has been published for the UK.

The HFEA publishes annual reports on IVF trends and figures. In 2018, it published the Fertility Treatment 2014–2016 Trends and Figures, which stated that “In 2016, the per embryo transferred birth rate for PGD (preimplantation genetic diagnosis) treatment cycles was 30%, compared to 21% for IVF overall, and 36% for frozen rate per embryo transferred, compared to 22% for IVF overall” (HFEA, Fertility treatment 2014–2016 Trends and figures [14]). Unfortunately, this report termed all PGT cycles “PGD” and did not differentiate between PGT-A and PGT-M/SR. In 2019, the HFEA Fertility Treatment 2017: Trends and Figures report noted the national incidence of PGD/T use but without any outcome data. The latest HFEA annual data, published in 2020, Fertility Treatment 2018: Trends and Figures, gives no PGT data.

Clear data on the use and effectiveness of PGT-A in clinics throughout the UK is therefore somewhat lacking. In order to examine the latest UK PGT-A outcomes, the authors requested from the HFEA via the Freedom of Information Act (FoI), the outcomes of PGT-A and non PGT-A cycles from the latest HFEA data collection.

## Methods

It is mandatory for all treatment cycles carried out at an ART centre and their subsequent outcomes to be reported to the HFEA in the UK. It should be noted that cycle parameters including, but not limited to, full patient demographics, numbers of eggs at collection, previous cycle outcomes, previous infertility history, previous biochemical loss, spontaneous abortion rate, preterm rates, twinning rates were not available to this study.

Several freedom of information (FoI) requests were made to the HFEA between 13th July 2020 and 22nd September 2020. These requests were intended to ascertain the PGT-A live birth occurrences in line with the 2018 HFEA annual report. We requested live births following ART cycles including PGT-A and not including PGT-A (non-PGT-A) between 2016 and 2018. The data was supplied on a per cycle started and per embryo transferred basis, broken down by maternal age, excluding surrogacy cycles, egg donation cycles and any cycle that had PGT-M or PGT-SR, including cycles where no embryos were transferred. It should be noted that we requested that all cycles where at least one oocyte was collected were included, any cycles started that did not reach egg collection or did not have any eggs collected were excluded. The transfers include fresh and frozen embryo transfers resulting from this original egg collection. Therefore, more than one embryo transfer event could occur per egg collection cycle.

The HFEA defines IVF cycle live birth rate (LBR) outcomes according to:

Live birth rate (LBR) per embryo transferred—the number of births divided by the sum of embryos transferred for treatment cycles starting in that year.

Live birth rate (LBR) per treatment cycle—the percentage of treatment cycles started in that year that resulted in a live birth.

HFEA outcome data is defined according to the year which the cycle started not the year of outcome, but it can be assumed that all PGT-A laboratory tests were performed in 2016–2018 and at this point all ISO15189 accredited PGT-A providers in the UK were utilising PGT-A via next-generation sequencing (NGS) technology. Hence, it can be assumed here that the reported outcomes in this dataset were from PGT-A using mainly NGS.

Since NGS technology cannot usually be used for fresh embryo transfer due to protocol time limitations, it is assumed that the majority of cycles using PGT-A resulted from frozen embryo transfers (although a small proportion recorded fresh PGT-A). At this time point, approximately 25 IVF clinics were licensed to provide PGT-A (CooperGenomics internal data); thus, this data was from a wide spectrum of the total of approximately 90 IVF clinics in the UK.

It should also be noted that over this time period, one individual patient could achieve multiple live birth events by returning to have a subsequent frozen embryo transferred, conventional reporting of outcomes will focus on achieving a single live birth event. Additionally, monozygotic twinning can occur following the transfer of a single embryo; these events have not been detailed in the data, therefore will represent 2 live birth events instead of 1. It has been reported that around 2.5% of single blastocyst embryo transfers will result in a twin pregnancy [15].

Throughout the results and discussion, we will refer to the following:

Cycles: Fresh egg collection resulting in one or more egg collected.

Fresh transfer: Embryo transferred directly following a fresh egg collection cycle.

Frozen transfer: Embryo transferred after being frozen and then thawed.

Statistical analysis was carried out using Statistical Package for the Social Science database (SPSS version 24) (SPSS, Chicago, IL, USA). A binary logistic model assuming binomial errors and employing a logit link function was applied to the data to compare live birth and outcomes per age group in PGT-A and non-PGT-A cycles. Odds ratios (OR) were calculated in the same way and presented with their 95% confidence intervals (CIs), presented in supplementary table. When possible, data were presented as percentages with 95% CIs. A *P* value of < 0.05 was considered statistically significant.

## Results

In the time period 2016–2018, a total of 2464 PGT-A cycles were reported out of 190,010 treatment cycles. PGT-A therefore represented 1.3% of all treatment cycles, an increase from the 0.6% of 2014–2016 cycles reported by Theobald et al. [11]. According to the HFEA Fertility treatment 2018: trends and figures [14] document, approximately two-thirds of non-PGT-A cycles and less than 5% of PGT-A cycles resulted in fresh embryo transfers, with the vast majority of PGT-A (and around one-third of regular IVF) being frozen embryo transfers. The document indicates LBR per embryo transferred is 2–3% higher for frozen compared to fresh embryo transfers.

**Fig. 1:**
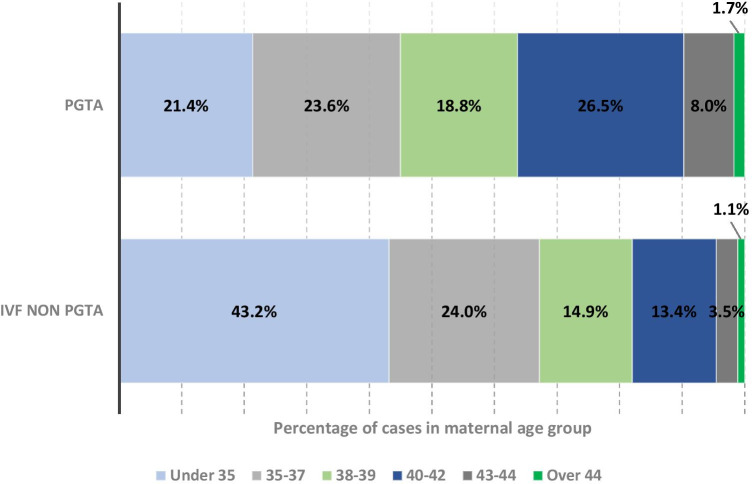
Distribution of PGTA and non-PGTA cases by maternal age group, shown as a percentage. Total number of PGT-A cases = 2464; total number of IVF non-PGT-A = 187,546

Figure 1 shows the percentage of PGT-A and non-PGT-A cycles distributed by maternal age and indicates a figure somewhat consistent across age groups ranging from 19 to 26%. In comparison, a far higher proportion of non-PGT-A cycles were in the < 35-year maternal age group (43% of all non-PGT-A cycles vs 21% of PGT-A cycles); however, the situation changes such that for maternal age over 38 to 44 years, the proportion of cycles utilising PGT-A is greater than for non-PGT-A cycles (% of PGT-A cycles vs % of non-PGT-A cycles; 19% vs 15% 38–39 years; 26% vs 13% 40–42 years; 6% vs 3% 43–44 years) to the point where PGT-A was most often used by the 40–42-year maternal age group.

**Fig. 2:**
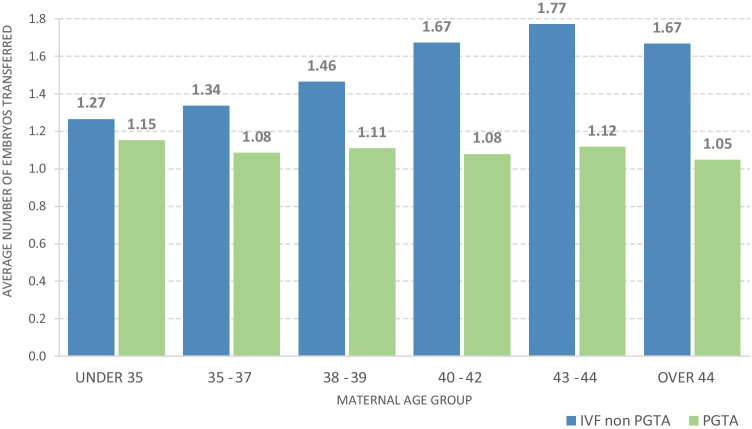
Average number of embryos transferred per cycle which achieved embryo transfer

We also examined the number of embryos transferred per cycle (Fig. 2), where 1.0 would indicate single embryo transfer for all cycles per age group. The number of embryos transferred per cycle for PGT-A cycles was consistent over all age groups, being an average of 1.1 (1.048–1.153). In contrast, the number of embryos transferred per non PGT-A cycle was consistently higher than PGT-A cycles; from 1.3 for maternal age < 35 years to 1.7 in 43–44-year age group. The proportion that was, in fact, double embryo transfers or multiple single embryo transfers is not available in this study. We can however conclude that more embryos are being transferred in the non-PGT-A group, as would be expected. Twinning rate outcomes for PGT-A were not available for this study. The HFEA Fertility Treatment 2018: Trends and Figures [14] reports an 8% twinning birth rate for all transfers, but the data is not available in this study to determine what proportion of PGT-A cycles resulted in multiple births.

**Fig. 3:**
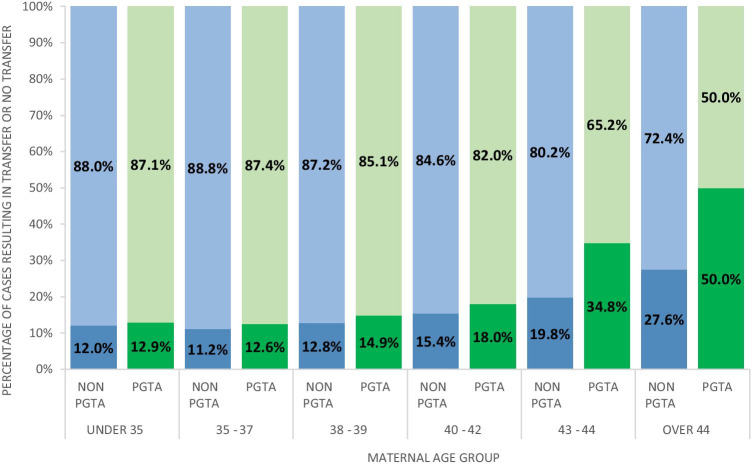
Percentage of cycles which had no embryos transferred vs embryo transfer for PGTA and non-PGTA by age group. (blue = non-PGTA; green = PGTA; top bar = transfer; bottom bar = no transfer)

Aneuploidy is widely reported to increase with maternal age and hence it is assumed that as maternal age increases, PGT-A cycles will deselect more aneuploid embryos for subsequent transfer [16]. This data is presented in Fig. 3. As expected, the percentage of cycles with no embryos transferred, which in most instances will be due to no embryos being suitable for transfer following an aneuploid result (although other factors such as spontaneous pregnancy and relationship breakdown could contribute to these figures), increased with maternal age. That is, in PGT-A cycles, it increased from a low of 12.5% of cycles in the 35–37-year group to 50% in the > 44-year group. This was in contrast to a low of 11.2% in the 35–37-year non-PGT-A group to a high of 27.6% of all non-PGT-A cycles in the > 44-year group. Somewhat surprisingly, there were no significant differences between the PGT-A and non-PGT-A groups in the < 35-year to 40–42-year groups (< 35: *P* = 0.546; 35–37: *P* = 0.284; 38–39: *P* = 0.182; 40–42: *P* = 0.070).

**Fig. 4:**
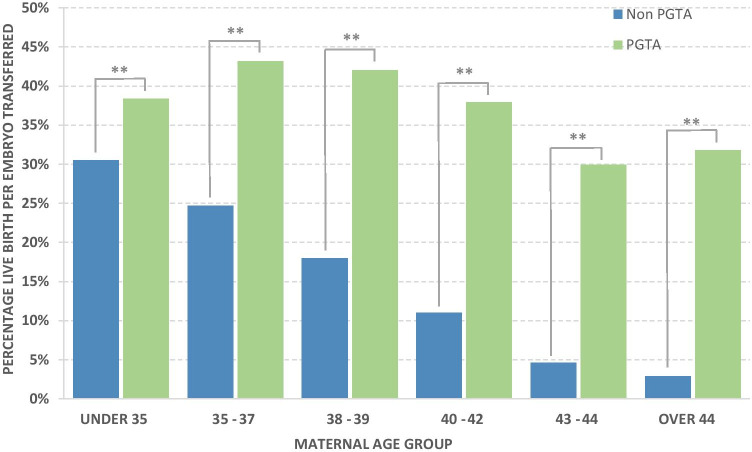
Percentage of live births per embryo transferred with and without PGTA, *P* values following binary logistic model, ** =  < 0.001

Live birth per embryo transferred (PET) for PGT-A and non-PGT-A is reported in Fig. 4. This data shows the highest LBR per embryo transferred of 43.2% in the 35–37-year maternal age group and, after this point, a decrease in LBR per embryo transferred to a low of 29.9% in the 43–44-year group. The > 44-year age group reported a slightly higher LBR per embryo transferred of 31.8%, although it should be noted that only 22 embryos were transferred in this age group. The percentage LBR per embryo transferred for the PGT-A cases was significantly higher (*p* < 0.05) for all age groups than for non-PGT-A groups (Supplementary data Table S1). The smallest difference was in the < 35-year group where there was a 7.9% difference between PGT-A and non-PGT-A LBR per embryo transferred. As maternal age increases in non-PGT-A cycles, there was a consistent drop-off in LBR per embryo transferred. The difference between LBR per embryo transferred outcomes was such that in the 40–42-year maternal age group (the group with the highest percentage of PGT-A cycles), there were 578 embryos transferred for 219 live births for the PGT-A group whilst in the non-PGT-A group, there were 35,484 embryos transferred for 3887 live births or 2.6 embryos transferred per live birth in the PGT-A group compared to 9.1 embryos transferred per live birth in the non-PGT-A group. The under 35-year age group showed the smallest difference in success rate of PGT-A vs non-PGT-A. That is, 2.6 and 3.3 embryos transferred respectively for each live birth and the > 44 years showed the greatest difference in success rate of PGT-A vs non-PGT-A, where 3.1 and 35.0 embryos transferred respectively for each live birth.

**Fig. 5:**
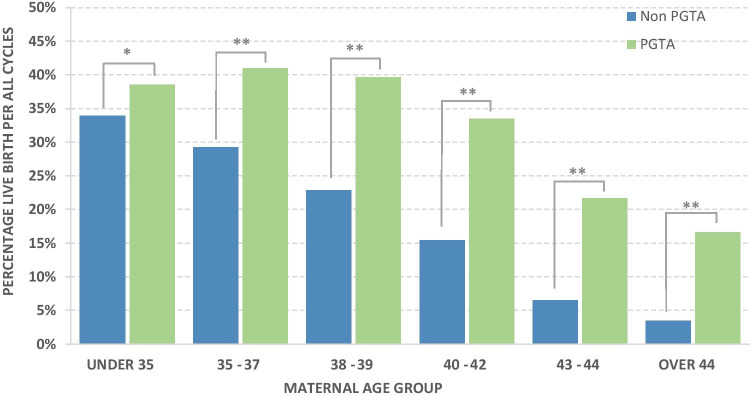
Percentage of live births per cycle with and without PGTA, *P* values following binary logistic model, * = 0.026, ** =  < 0.001

LBR per treatment cycle is also included in Fig. 5 (including cycles that did not result in an embryo transfer). For all age groups, LBR per treatment cycle was significantly higher (*p* < 0.05) for PGT-A compared to non-PGT-A (Supplementary data Table S1). We note that most groups have a higher percentage of live births per treatment cycle than per embryo transferred; this reflects those cycles which resulted in multiple embryos transferred and/or multiple single embryo transfers. Compared to LBR per embryo transferred, for LBR per treatment cycle, there was a marked trend towards lower LBR with increasing maternal age over 38–39 years for PGT-A cycles such that LBR per treatment cycle was 39.6% in 38–39-year group, declining to 16.7% in > 44-year group. LBR per treatment cycle for non-PGT-A cycles, as seen in LBR per embryo transferred, declined with maternal age and in all instances was significantly lower for each age group than PGT-A LBR per treatment cycle.

## Discussion

The HFEA has a rigorous process for determining whether treatment options outside “routine IVF treatment”, termed “add-ons”, are to be recommend to patients [17]. This process looks to stringent evidence-based medicine approaches, specifically only taking into consideration the outcomes of multi-centre peer-reviewed double-blinded randomised control trials (RCTs). The findings of the analysis of each add on is then distilled down to a patient-friendly “traffic light” system (note UK traffic lights show three colours: green to go, amber to slow down and red to stop). At the current time PGT-A is “red” and therefore described as “an add-on where there is no evidence to show that it can improve live birth rate” and “for an add-on where there is evidence to show that the add-on is unsafe” (HFEA, 2020 [18]).

At the present time, only one PGT-A multi-centre peer-reviewed double-blinded randomised control trial (RCT) has been published, the STAR trial [19] that incorporated NGS-based PGT-A technology on biopsied blastocysts with subsequent embryo vitrification/thawing/transfer, or, in other words, the technology that is currently used in UK PGT-A cycles. This trial showed no benefit in ongoing pregnancy rate (OPR 20 weeks) for each cycle started for PGT-A vs non-PGT-A. A post hoc analysis of maternal age 35–40 years however showed a significant increase in OPR per embryo transfer (51% vs. 37%) of PGT-A vs non-PGT-A cycles; this was controversial, however, and not considered as reliable evidence by the HFEA Scientific and Clinical Advances Advisory Committee (SCAAC), which provides recommendations on traffic light indications for each add-on. There are 5 other PGT-A RCTs currently published that have utilised PGT-A technologies, which report on all 24 chromosomes, all but the ESHRE ESTEEM trial (involving polar body biopsy, which was not conducted in the 2016–2018 timeframe reported here) have attracted some criticism due to them being underpowered, showing selection bias, having poor blinding of participants or not being multi-centre and have been excluded from meta-analysis [5]. Although not an RCT, we would however consider the multi-centre, prospective, blinded, PGT-A non-selection trial of Tiegs et al. [20] to be worthy of consideration when looking at the efficacy of PGT-A. In this study, 402 patients were included, after trophectoderm biopsy, 484 single, frozen, blastocyst transfers, blinded to their PGT-A results. Of the group of 102 embryo transfers reported to be aneuploid, no (0%) sustained implantation nor live births were reported and in the group of 312 embryo transfers reported to be euploid 64.7% reported a sustained implantation or live birth, demonstrating the high predictive power of PGT-A. Hence, very few viable embryos are discarded when they are reported aneuploid, often a criticism of PGT-A.

The authors here subscribe to the view that multi-centre double-blinded RCTs are the highest form of study design for any adjunct treatment to be considered for use in ART. We do not however feel that it is the only source of evidence that should be used to inform patients of their reproductive choices. The design of a study gives no indication to the quality in which it is performed, and the results of even multi-centre studies give only limited indications of how transferrable the technology is from clinic to clinic. PGT-A is in clinical use in the UK and many other countries, giving us the ability to address the question of the efficacy of PGT-A “in the real world”—i.e. across all centres in the UK performing it. What this study lacks in the ability to randomise the patient groups, it compensates for in the sheer numbers reported, and provides additional information about transferability (across an estimated 25 centres nationwide).

We nonetheless acknowledge there are some limitations with the data recorded by the HFEA with regard to PGT-A cycles, similarly to those noted by Theobald et al. [11]. For instance, some patients, especially in the older age categories, may have intended to undertake PGT-A as part of their treatment, but they may have encountered failed fertilisation, embryo arrest, failed blastulation or embryos being of too poor a quality to perform biopsy. In these situations, it is unlikely that the cycles intent to undergo embryo biopsy and subsequent PGT-A analysis may not have been recorded. The cycles reported by the HFEA, where no embryos were transferred for the PGT-A group, may simply represent those patients which only had aneuploid embryos reported following genetic analysis. Additionally, operator errors can and do occur, where some PGT-A cycles may erroneously be recorded as PGT-M and PGT-SR or indeed not have the genetic testing recorded at all. Some clinics additionally batch embryos for biopsy, therefore providing an artificially high rate of obtaining a euploid embryo available for transfer per cycle undergoing PGT-A. The cycles to “batch” embryos prior to the cycle in which biopsy and genetic testing is performed may likely be recorded as “for embryo storage”, therefore would not have been captured within this dataset. We also note that when embryos are thawed, biopsied and re-frozen, this is not required to be reported to the HFEA, therefore missing such cycles which result in all aneuploid embryos. Moreover, a PGT-A case that did not result in a transfer may be because it did not survive the vitrification/warming or was of poor quality that it did not recover. We did not request details of cycles which were cancelled prior to oocyte retrieval, and as such, it would be anticipated that for older patients more cycles would be cancelled prior to this stage. Future studies will, no doubt, be able to address, and control for, these confounding factors. Despite this, the levels of statistical significance seen on all age groups, but particularly in the older age groups, suggest that the observed effects are real, despite the possible confounding factors. Our findings show that PGT-A is used roughly evenly across all age groups from < 35 to 40–42 years, reducing only in the > 43s. This is in contrast to non-PGT-A cycles, which are most frequently used for the < 35-year maternal age group. No maternal age-related increase in embryos per transfer was observed however.

The number of embryos transferred per PGT-A cycle is much closer to one embryo transferred than for non-PGT-A cycles indicating potential health benefits for PGT-A in avoiding the obstetrics complications associated with multiple births. We were however not able to obtain the multiple births (twins, triplets etc.) per transfer data for the PGT-A cycles and we therefore could not determine directly whether PGT-A cycles had a lower or higher multiple birth rate per embryo transfer. Given that PGT-A cycles had an average number of embryos transferred per cycle of 1.1, closer to the single embryo transfer ideal of 1.0, it still would result in 1 in 11 cycles having a double embryo transfer (DET). A proportion would still have resulted in some multiple births but presumably much lower than the non-PGT-A group in any age category. It is also not possible to identify how many transfers were multiple SETs or returned for a second embryo transfer following a live birth event. Therefore, there is potential for a single cycle to have yielded multiple singleton live births and consequently more cycles to have achieved no live births which is not captured in this data. There is no evidence that these would have been disproportionately represented in any group however.

Part of our FoI request pertaining to the number of cycles where no embryo had been transferred is related to the well-documented fact that aneuploidy increases with maternal age. Aneuploidy levels range from less than 40% for < 35 years to over 80% in > 44-year age group [21]. The high level of aneuploidy with advanced maternal age is purported to be a major limitation of any PGT-A protocol’s ability to increase LBR per cycle started since high levels of aneuploidy will lead to cycles with no embryos for transfer [22]. In this dataset, the percentage of PGT-A cycles where no embryos were transferred increased with maternal age from 12.6% in 35–37-year age group to 50% in the > 44-year age group. However, this is less than the 12.7% of cycles recorded with no embryo for transfer in the STAR trial, albeit with an average maternal of 33.7 years. In the current study, there were no significant differences in the proportion with no embryo for transfer of PGT-A vs non-PGT-A cycles up until the 43–44-year age group (34.8.0% PGT-A vs 19.8% non-PGT-A). The number of PGT-A cycles with no embryo for transfer reported here, particularly in the older age groups, could be due to a number of factors: Increase in mosaic diagnosis with NGS whereas previously using array comparative genomics hybridisation (aCGH) protocols these would have been reported as aneuploid. According to CooperGenomics internal data, 14% of embryos were reported as mosaic during this time period. This altered reporting strategy could potentially produce a higher percentage of embryos that could be considered for transfer. Additionally, cycles designated for PGT-A by clinics and with no embryo for transfer could possibly be recorded in the HFEA system as non-PGT-A rather than the correctly assigned to a “PGT-A with no embryos transferred” group.

The finding that LBR per embryo transferred is similar in all age groups following PGT-A has been previously reported [23]. That is, there is evidence that PGT-A mitigates the maternal age effect. Although in the present study, we did not feel the need to review these findings further, this study clearly demonstrates that fewer embryos (2.6 per cycle on average) are required to achieve a pregnancy following PGT-A compared to regular IVF (3.3. per cycle). Moreover, this differential becomes greater with increasing maternal age. This information is of particular interest in the UK since the HFEA uses LBR per embryo transferred in their “Choose a clinic” (www.hfea.gov.uk/choose-a-clinic/clinic-search/) search facility for prospective IVF patients. Additionally, it should be noted that the majority of PGT-A cycles reported here would have resulted in all embryos being frozen and subsequent frozen embryo transfer. This may have influenced the differences in our comparisons of LBR to some degree, as the latest HFEA data comparing fresh vs frozen embryo transfers (Fertility treatment 2018: trends and figures [14]) indicates LBR per embryo transferred 2–3% higher for frozen compared to fresh embryo transfer cycles. Despite this, however, a 2–3% difference is, in no way, sufficient enough of a confounding factor to explain the observed higher LBR per embryo transferred as a result of PGT-A.

Theobald et al. [11] compared LBR per treatment cycle for under and over 38 years, showing an annually increasing LBR for frozen PGT-A transfers under 38 years of 28.0%/33.0%/39.5% (2014/2015/2016) and for over 38 years of 25.8%/30.2%/34.3% (2014/2015/2016). This trend of LBR per treatment cycle was observed to continue in our current dataset (Fig. 5), with 39.8% for the under 38 years and 33.3% for the over 38 years. It has been previously reported that NGS-based PGT-A results in an improved LBR per embryo transferred and per “intention to treat” compared to aCGH. From around 2014, PGT-A testing in the UK migrated from aCGH to NGS, which may be part of the explanation as to why LBR per treatment cycle showed an increase in success rate over this period [24]. Another explanation may however be increasing experience and expertise of UK IVF labs with associated protocols such as biopsy and vitrification.

In this dataset, LBR per treatment cycle was significantly improved as a result of PGT-A in all age groups (Fig. 5). Somewhat unexpectedly, this improvement was also seen in the youngest (under 35) age group. Post hoc subgroup analysis in the STAR trial (which had a similar number of participating clinics) did not show such an effect and the differences may be due to the absence of proper randomisation in the current study leading to one of the groups being disproportionately represented. That is, although the patients were matched by age, other factors were not taken into account. Conceivably, clinics running PGT-A could have higher success rates than those clinics that do not and this underlying increased success rates could have skewed the data towards higher PGT-A success rates. However, without further data per clinic, it is not feasible to determine if this was a confounding factor in this cohort. Haviland et al. [25] reported a retrospective analysis of a cohort of 8227 cycles from a single US ART centre, where they were able to examine the demographic and clinical characteristics to enable them to use a propensity score to fully match 1015 women who had PGT-A (aCGH and NGS) with 1015 non PGT-A women. They reported very similar levels of cycles with no embryo for transfer in PGT-A cycles to this study, but higher LBR per “intent to treat” in all groups. Improvement in the under 35s is also consistent with a small number of retrospective studies [7, 10]. Also, like the STAR trial and this study, they noted a higher LBR per cycle in the older age groups, albeit with higher LBR than reported in the UK HFEA data. An alternative explanation is of course that in recent years clinics performing PGT-A now have enough expertise to reduce potential damage caused by the biopsy process that a benefit can be seen in the younger (< 35) age groups also.

Whilst this study does not fulfil the HFEA’s self-imposed criteria for “traffic light” inclusion, it nonetheless represents the largest reported cohort in the UK of the efficacy of PGT-A. It also contains its own published data. The issue of matching PGT-A to non-PGT-A groups has been mentioned already; however, it does nonetheless indicate that there may be many potential benefits of PGT-A.

## Conclusions

Our data, obtained from the HFEA’s database, provide compelling evidence of the efficacy of PGT-A in terms of resulting in a higher percentage of LBR per treatment cycle started, and per embryo transferred. Although it does result in a smaller number of embryos transferred, this, in turn, can lead to lower multiple live births (where one embryo is transferred instead of two), and lower rates of failed embryo transfer events (higher implantation rates and/or lower biochemical pregnancy loss rates and/or lower miscarriage rates). This is particularly evident in the oldest age groups. As the patients were not randomised, the figures do not qualify for consideration by the HFEA, despite similar figures being used by them for other purposes, e.g. ranking ART clinics. The potential for further expanding this study to examine the impact of demographic and clinical characteristics is dependent on additional data collected and reported by the HFEA. We therefore urge for a review of the required data to be collected from licensed clinics by the HFEA so that questions arising from the limitations of this study can be answered and a more complete analysis on the impact of PGT-A cycles and, in turn, non-PGT-A cycles on LBR per treatment cycle.

## Supplementary Information

Below is the link to the electronic supplementary material.

Supplementary file1 (DOCX 19.3 KB)
