# Supplementary material for: Analysis of IVF live birth outcomes with and without preimplantation genetic testing for aneuploidy (PGT-A): UK Human Fertilisation and Embryology Authority data collection 2016–2018
Source: J Assist Reprod Genet. 2021 Nov 12;38(12):3277–85. doi: 10.1007/s10815-021-02349-0 (PMC8666405; doi:10.1007/s10815-021-02349-0)
Supplement: Supplementary file 1 — Supplementary file1 (DOCX 19.3 KB) [file 10815_2021_2349_MOESM1_ESM.docx]

Supplementary information

| **Age group** |  | **Non PGT-A** | **PGT-A** | ***P* Value** | **OR (95% CI)** |
| --- | --- | --- | --- | --- | --- |
| **< 35** | **Total cycles** | **80971** | **527** |  |  |
|  | Live birth PET | 27449/90097 (30.5%) | 203/529 (38.4%) | <0.001 | 1.42 (1.19-1.69) |
|  | Live birth PTC | 27449/80971 (33.9%) | 203/527 (38.5%) | 0.026 | 1.22 (1.02-1.46) |
|  |  |  |  |  |  |
| **35-37** | **Total cycles** | **45014** | **581** |  |  |
|  | Live birth PET | 13174/53394 (24.7%) | 238/551 (43.2%) | <0.001 | 2.32 (1.96-2.75) |
|  | Live birth PTC | 13174/45014 (29.3%) | 238/581 (41.0%) | <0.001 | 1.68 (1.42-1.98) |
|  |  |  |  |  |  |
| **38-39** | **Total cycles** | **27895** | **462** |  |  |
|  | Live birth PET | 6388/35560 (18.0%) | 183/436 (42.0%) | <0.001 | 3.30 (2.73-4.00) |
|  | Live birth PTC | 6388/27895 (22.9%) | 183/462 (39.6%) | <0.001 | 2.21 (1.83-2.67) |
|  |  |  |  |  |  |
| **40-42** | **Total cycles** | **25102** | **654** |  |  |
|  | Live birth PET | 3887/35484 (11.0%) | 219/578 (37.9%) | <0.001 | 4.96 (4.18-5.89) |
|  | Live birth PTC | 3887/25102 (15.5%) | 219/654 (33.5%) | <0.001 | 2.75 (2.33-3.24) |
|  |  |  |  |  |  |
| **43-44** | **Total cycles** | **6471** | **198** |  |  |
|  | Live birth PET | 421/9190 (4.6%) | 43/144 (29.9%) | <0.001 | 8.87 (6.13-12.84) |
|  | Live birth PTC | 421/6471 (6.5%) | 43/198 (21.7%) | <0.001 | 3.99 (2.80-5.67) |
|  |  |  |  |  |  |
| **> 44** | **Total cycles** | **2093** | **42** |  |  |
|  | Live birth PET | 72/2524 (2.9%) | 7/22 (31.8%) | <0.001 | 15.89 (6.29-40.17) |
|  | Live birth PTC | 72/2093 (3.4%) | 7/42 (16.7%) | <0.001 | 5.61 (2.41-13.07) |

Supplementary table S1. Live births per embryo transferred (PET) and per cycle started (PTC) for each maternal age group
